# *Colletotrichum gloeosporioides* Cg2LysM contributed to virulence toward rubber tree through affecting invasive structure and inhibiting chitin-triggered plant immunity

**DOI:** 10.3389/fmicb.2023.1129101

**Published:** 2023-02-17

**Authors:** Li Zhao, Zhiwen Liao, Liping Feng, Bang An, Chaozu He, Qiannan Wang, Hongli Luo

**Affiliations:** ^1^School of Life Sciences, Hainan University, Haikou, China; ^2^College of Tropical Corps, Hainan University, Haikou, China; ^3^Sanya Nanfan Research Institute of Hainan University, Hainan Yazhou Bay Seed Laboratory, Sanya, China

**Keywords:** *Colletotrichum gloeosporioides*, Cg2LysM, pathogenesis, chitin binding, rubber tree, plant immunity

## Abstract

Fungal chitin, as a typical microorganism-associated molecular pattern (PAMP), was recognized by plant LysM-containing protein to induce immunity called pattern-triggered immunity (PTI). To successfully infect host plant, fungal pathogens secreted LysM-containing effectors to inhibit chitin-induced plant immunity. Filamentous fungus *Colletotrichum gloeosporioides* caused rubber tree anthracnose which resulted in serious loss of natural rubber production worldwide. However, little is known about the pathogenesis mediated by LysM effector of *C. gloeosporioide*. In this study, we identified a two LysM-containing effector in *C. gloeosporioide* and named as Cg2LysM. Cg2LysM was involved not only in conidiation, appressorium formation, invasion growth and the virulence to rubber tree, but also in melanin synthesis of *C. gloeosporioides*. Moreover, Cg2LysM showed chitin-binding activity and suppression of chitin-triggered immunity of rubber tree such as ROS production and the expression of defense relative genes *HbPR1*, *HbPR5*, *HbNPR1* and *HbPAD4*. This work suggested that Cg2LysM effector facilitate infection of *C. gloeosporioides* to rubber tree through affecting invasive structure and inhibiting chitin-triggered plant immunity.

## Introduction

Plant diseases occur from the interaction between host plants and pathogens. Based on plant innate immunity theory, the recognition of conserved microbial surface structures termed ‘microorganism-associated molecular patterns’ (PAMPs) by cell surface pattern recognition receptors (PRRs) to elicit pattern-triggered immunity (PTI; Jones and Dangl, 2006). PTI confers full immunity to host non-adapted pathogens and partial immunity to host-adapted pathogens (Wang et al., 2022). To successfully infect host plant, the latter pathogens have evolved ways to evade PTI by secreting effectors into plant cells, which was termed effector triggered susceptibility (ETS). In turn, plants have evolved resistance (R) proteins, which recognize effectors and led to effector triggered immunity (ETI) which was mostly characterized by a hypersensitive response (HR; Jones and Dangl, 2006; Stergiopoulos and de Wit, 2009). These interactions are complex, not only involving in multifaceted recognition of pathogens by the plants but also involving in subtle evasion from the pathogens (Campos et al., 2021). In plants, there are a large repertoire of genes encoding cell surface and intracellular immune receptors for various danger signals detection to active effective defenses against pathogen infection. However, this also drives pathogens to evolve mechanisms of plant immune recognition evasion to facilitate colonization, such as evasion of PRR-mediated detection though altering, sequestering, degrading or preventing release of MAMPs (Wang et al., 2022).

Lysin motif (LysM) was originally identified as a protein domain in a *Bacillus* phage lysozyme that degraded bacterial cell walls (Garvey et al., 1986). So far, a large number of LysM-containing proteins were found in eukaryotes including plants and fungi (Buist et al., 2008). As receptors, plant LysM-containing proteins could recognize chitin, a well-known PAMP released from fungal cell walls during infection, to trigger PTI (Miya et al., 2007; Kombrink et al., 2011; Thomma et al., 2011; Sanchez-Vallet et al., 2015). However, many fungal pathogens could secrete LysM-containing effectors similar to plant’s chitin receptors (Hu et al., 2021), sequestering and masking fungi to prevent fungal cell wall degradation by plant chitinases (Kombrink and Thomma, 2013; Sanchez-Vallet et al., 2015; Volk et al., 2019; Gong et al., 2020), and inhibiting chitin-induced plant immunity by blocking chitin sensing or signaling (Bolton et al., 2008; Jonge et al., 2010; Marshall et al., 2011; Mentlak et al., 2012; Lee et al., 2014; Takahara et al., 2016; Kombrink et al., 2017; Dolfors et al., 2019; Tian et al., 2021). *Cladosporium fulvum* Ecp6 was a typical LysM protein with three LysM domains and could chelate chitin during infection (Bolton et al., 2008). During infection, Ecp6 sequesters chitin oligosaccharides released from the cell walls of invading hyphae to prevent chitin mediated PTI (Jonge et al., 2010). In contrast to *C. fulvum* Ecp6, *Mycosphaerella graminicola* Mg1LysM and Mg3LysM also protected fungal hyphae against plant-derived hydrolytic enzymes, however, only Mg3LysM blocked the elicitation of chitin-induced plant defenses (Marshall et al., 2011). Mg3LysM deletion mutant showed severely impaired ability in leaf colonization and lesion formation, and was nonpathogenic toward the wheats silenced either CERK1 or CEBiP which were required for activation of chitin-induced defenses as the receptor-like proteins in wheat (Marshall et al., 2011; Lee et al., 2014), indicating the importance of Mg3LysM for fungal evasion of PTI through blocking chitin signaling. The LysM-containing effectors, such as Slp1 in *Magnaporthe oryzae*, ChELP1 and ChELP2 in *Colletotrichum higginsianum*, Vd2LysM in *Verticillium dahliae*, RsLysM in *Rhizoctonia solani*, Mgx1LysM in *Zymoseptoria tritici*, had been proved to contribute to virulence by disturbing the activation of chitin-triggered immunity (Mentlak et al., 2012; Takahara et al., 2016; Kombrink et al., 2017; Dolfors et al., 2019; Tian et al., 2021). The evolutionary study from 57 endophytes and fungi with diverse lifestyles showed that LysM effectors contributed to fungal lifestyle (Suarez-Fernandez et al., 2021). Additionally, RiSLM, a secreted LysM effector from arbuscular mycorrhiza (AM) fungal species *Rhizophagus irregularis*, protected fungal cell walls from chitinase by binding to chitin oligosaccharides and effectively interfere with chitin-triggered immune response to subvert chitin-triggered immunity during symbiosis (Gust et al., 2012; Zeng et al., 2020). Similarly, the beneficial fungus *Trichoderma viride* employed a LysM effector Tal6 to sequester GlcNAc oligomers, thereby interfering with the perception of fungal-derived N-acetylglucosamine by plants surveillance machinery leading to protection of fungal hyphae from the host’s chitinases (Romero-Contreras et al., 2019).

Some evidences had demonstrated that fungal LysMs were not always only involved in manipulation of plant immunity, but also in the self-regulation of fungal growth and development. *Trichoderma atrovirideis* LysM protein TAL6 was shown to specifically inhibit germination of *Trichoderma* spp. rather than fungal-plant interactions (Seidl-Seiboth et al., 2013). PeLysM3 was identified from *Penicillium expansum* and contained one LysM domain, which null mutant exhibited slightly lower rate of radial growth, significantly lower percent of germinating spores and shorter germ tubes (Levin et al., 2017), but *Verticillium nonalfalfae* LysM protein VnaChtBP deletion had no significant effect on the growth and pathogenicity (Volk et al., 2019). In *M. graminicola*, the LysM effector Mg1LysM negatively regulated pycnidia formation on wheat, but Mgx1LysM and Mg3LysM positively regulated pycnidia formation (Tian et al., 2021).

In *Colletotrichum*, two LysM effectors ChELP1 and ChELP2 had been identified in *C. higginsianum.* Both ChELP1 and ChELP2 were essential for fungal virulence and appressorium-mediated penetration and suppressed the chitin-triggered activation of two immune-related plant mitogen-activated protein kinases (Takahara et al., 2016). *C. graminicola* also contains two homologs of Ecp6 which had been experimentally proven to interact with chitin and modulate plant immunity (Sanz-Martin et al., 2016). Natural rubber is an irreplaceable important strategic resource and industrial raw material and mainly produced from rubber tree (*Hevea brasiliensis*). The rubber tree anthracnose, caused by filamentous fungus *Colletotrichum gloeosporioides*, resulted in serious loss of natural rubber production worldwide (Liu et al., 2018; Wang et al., 2018). However, little is known about the pathogenesis of *C. gloeosporioides* toward rubber tree. To elucidate the pathogenic mechanism of *C. gloeosporioides*, the candidate genes encoding effectors were predicted in the genome of *C. gloeosporioides* and one of candidate effector containing two LysM was named Cg2LysM. In this study, the contribution of Cg2LysM in fungal virulence and development was investigated. These results enriched our understanding of the molecular pathogenesis mechanisms of *C. gloeosporioides* on rubber tree.

## Materials and methods

### Biological materials and growth conditions

*Colletotrichum gloeosporioides* was isolated from the leaves of *Hevea brasiliensis* with anthracnose. The wild type and mutant strains of *C. gloeosporioides* were grown on potato dextrose agar (PDA) at 28°C in the dark. *Hevea brasiliensis* (Reyan 7-33-97) plants were grown on soil at 28°C under natural light condition.

### Amplification and bioinformatics analysis of *Cg2LysM* gene

The nucleotide sequence of *Cg2LysM* was amplified by RT-PCR and confirmed by sequencing. The primers used for amplification of *Cg2LysM* were list in Supplementary Table 1. The amino acid sequence of *Cg2LysM* was deduced by DNAMAN software. Prediction of signal peptides was performed online using the SignalP 5.0 analysis tool.^1^ The conserved domains were predicted using the SMART website.^2^ The bootstrap neighbor-joining phylogenetic tree was constructed with MEGA 7.0 software.

### Generation of *Cg2LysM* knockout and complementary mutants

The knockout vector for Cg2LysM was designed as shown in Supplementary Figure S2A. The 5′ and 3′ flanking fragments of *Cg2LysM* in genome were amplified and ligated into the vector pCB1532, which carried the acetolactate synthase gene (SUR) cassette conferring resistance to chlorimuron-ethyl. The knockout vector was linearized with *Xma*I before transformation. Fungal transformation was carried out as described in our previous work (Wang et al., 2018). The knockout mutants were detected by two round PCR analyses for homologous integration of the 5′ and 3′ flanking fragments. For the complementary vector, the open reading frame of *Cg2LysM* fused with 3 × FLAG coding sequence was cloned into the vector harboring *ToxA* promoter, nos terminator and a hygromycin phosphotransferase gene (HPH). The linearized complementary vector was transformed into *Cg2LysM* knockout mutant which was detected by PCR analyses. The primers used for mutants’ diagnosis were list in Supplementary Table 1.

### Pathogenicity test

The detached leaves from rubber tree variety 7-33-97 were used for pathogenicity test at the “light green” stage. Conidia were harvested from the WT and mutant strains grown on PDA medium for 7 days, washed with double-distilled H_2_O, and resuspended in a solution of 5% Sabouraud Maltose Broth (Difco) to a final concentration of 2 × 10^5^ conidia/ml. Then droplets (5 μl) of the conidial suspensions were used to inoculate onto the wounded rubber tree leaves. The inoculated leaves were kept in a moist chamber at 28°C under natural illumination for 4 days. The disease symptoms were photographed and the lesions were measured. Each treatment contained three replicates, and the entire experiment was repeated three times. Statistical analysis was performed by SPSS software (version 20), with *p <* 0.05 as statistically significant.

### RNA isolation, cDNA synthesis and qRT-PCR

CTAB-LiCl method was used for fungal RNA extraction (Yang et al., 2020). Polysaccharide polyphenol plant total RNA extraction kit (Tiangen: DP441) was used for plant total RNA extraction. The contaminating DNA was eliminated using RNase-free DNase, and the first-strand cDNA was synthesized using Revert Aid First Strand cDNA Synthesis Kit (Thermo Fisher). Quantitative RT-PCR (qRT-PCR) analysis was performed with SYBR Premix Ex Taq II (Takara, Dalian, China) using StepOne (Applied Biosystems) Real-time PCR instruments. The beta-tubulin-1 (*Cgβ-tub1*) gene and was used as an endogenous for normalization in *C. gloeosporioides* and 18S rRNA (*Hb18S*) gene was used as an endogenous control for normalization in *H. brasiliensis*. Specific amplification primers used in this research were shown in Supplementary Table 1. Relative expression levels of target genes were estimated using the 2^−ΔΔCt^ method.

### Fungal growth and conidiation assay

For the fungal growth assay, 5-mm-diameter disks of hypha were inoculated on PDA for 7 days, the colonial morphology was observed and colonial diameters were recorded for statistical analysis. For the conidiation assay, conidia were harvested from the strains growing on PDA medium for 8 days and inoculated into 50 ml liquid CMC medium to the final concentration of 10^4^/ml. Then all samples were cultured at 28°C with shaking (150 rpm) for 2 days, and the conidia numbers were calculated under a microscope. The experiments were repeated three times. Statistical analysis was performed by SPSS software (version 20), with *p <* 0.05 as statistically significant.

### Appressorium development assay

For appressorium development assay, 20 μl drops of conidial suspensions were placed on a plastic plate and incubated at 28°C. After 2, 4, and 6 h incubation, the conidial morphology was observed and the percentages of conidial germination and appressorium formation were determined under a microscope, respectively. The invasive growth assay was performed on onion epidermis which was sprayed with 2.5 × 10^5^/ml of conidia and placed on agar medium plate at 28°C for 12 h, and the invasion was observed under a microscope. The experiments were repeated three times, and at least 100 conidia were detected per replicate.

### Melanin content measurement

For the melanin content measurement, 5-mm-diameter of hypha disks were inoculated on PDA medium covered with cellophane and cultured for 7 days at 28°C, then the color of the colonies was observed and photographed. The melanin content was measured with the Fungal melanin quantification kit (GENMED SCIENTIFICS INC, United States). Briefly, mycelium was collected from the surface of cellophane by tweezers, and ground to a fine powder in liquid nitrogen. Then 0.1 g of the powder was transferred into a 1.5 ml tube, and the melanin was extracted according to the manufacture’s protocol. Melanin content was quantitated at 360 nm using a spectrophotometer (Eppendorf, Germany).

### Protein expression in mesophyll protoplasm of rubber tree

Rubber tree mesophyll protoplasm was prepared as previously reported protocol (Jones and Dangl, 2006; Zhang et al., 2016). 10 μg of recombinant plasmid DNA were introduced into a 200 μl aliquot of the protoplast solution (adjusted concentration to 2 × 10^6^/ml) was transferred to a new 1.5 ml microfuge tube, and equal volume (protoplast plus plasmid) of 40% PEG solution (containing 40% (W/W) PEG4000, 0.2 M mannitol, 0.1 M CaCl_2_) were added, and the mixed solution was incubated for 10–15 min at room temperature. The transformation reaction was diluted with four volumes of W5 solution and centrifuged at 200 *g* for 1 min to remove PEG as possible. The protoplasts were then gently suspended in WI solution (0.5 M mannitol, 4 mM MES, 20 mM KCl, pH 5.7) and kept under weak light overnight for Western blotting.

### Chitin binding assay

For chitin binding assay of Cg2LysM, the coding sequence of *Cg2LysM* was inserted into the transient expression vector pUC19-35S-Flag to generate recombinant plasmid pUC19-35S-Cg2LysM-Flag. pUC19-35S-Flag and pUC19-35S-Cg2LysM-Flag were expressed in rubber tree mesophyll protoplasm respectively, and the total protein extraction was referred to the method of Zhang et al. (2016). 0.5 ml protein solution with a concentration of 20 mg/ml for each sample was incubated with 50 μl magic chitin beads (New England Biolabs, Beverly, United States) at 4°C on a rocking platform for 4 h. The insoluble pellet fraction was collected on a magnetic stand and rinsed three times in CBD column buffer to remove unbounded protein. The pellet samples were detected by Western blotting with primary antibody anti-Flag antibody (1:10,000) and secondary FITC-conjugated goat antimouse antibody (1:5,000). Protein bands were detected using Amersham ECL Prime Western Blotting Detection Reagents in an ImageQuant LAS 4000mini.

### Reactive oxygen species measurement

ROS measurement was performed as previously reported protocol in rubber tree mesophyll protoplasm (Zhang et al., 2016). Mesophyll protoplasm expressing pUC19-35S-Flag and pUC19-35S-Cg2LysM-Flag were treated with or without 200 μg/ml chitin, respectively. ROS were measured every 10 for 600 min with the Reactive Oxygen Species Assay Kit (Beyotime Institute of Biotechnology, Haimen, China), following the manufacturer’s instructions. Fluorescence was read at 485 nm (excitation) and 530 nm (emission) with a fluorescence microplate reader (Bio-TEK, United States).

## Results

### Cloning and analysis of *Cg2LysM* in *Colletotrichum gloeosporioides*

**Figure 1:**
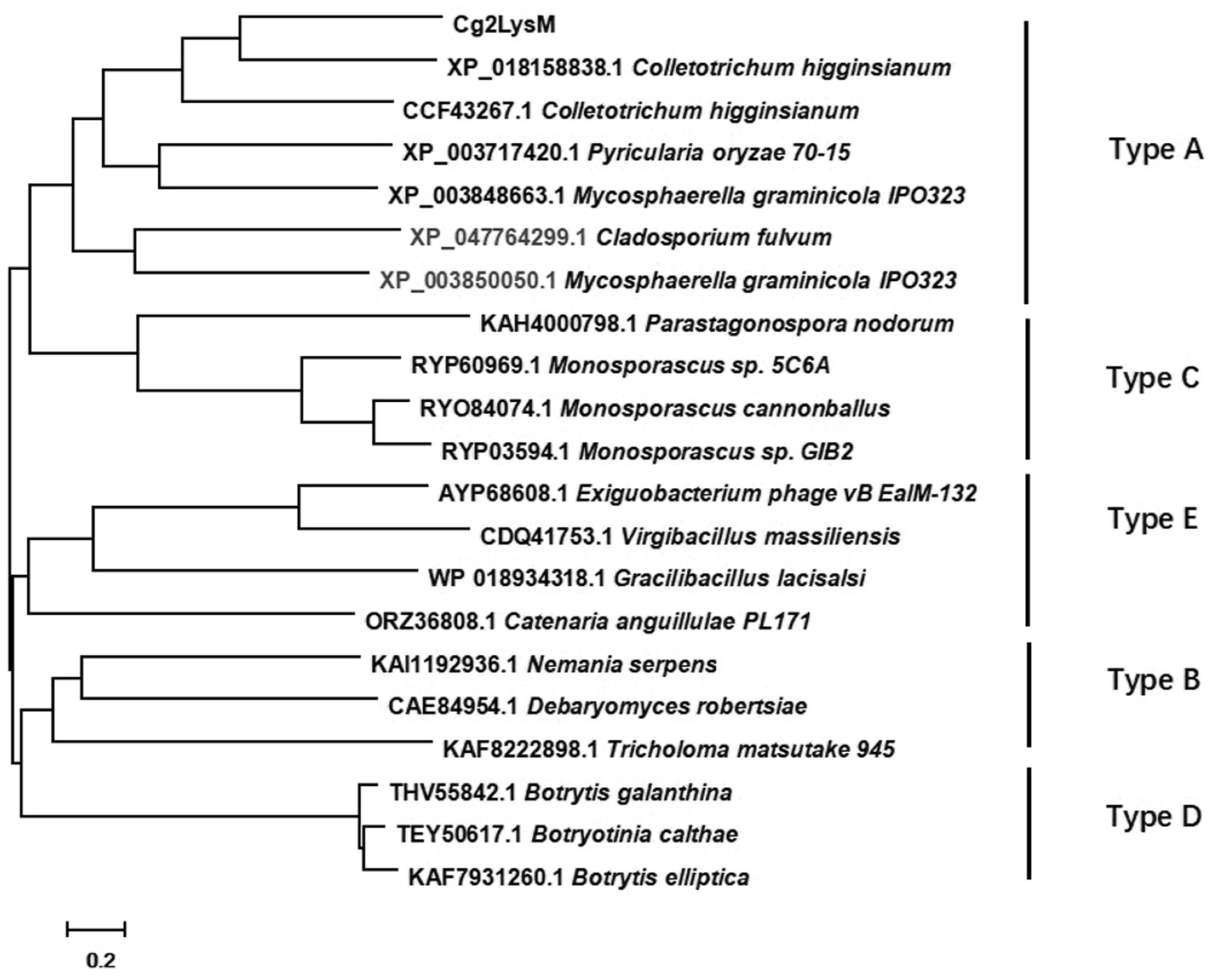
Phylogenetic analysis of Cg2LysM with different type of LysM effectors in fungi.

The genes encoding extracellular secretory proteins were predicted in genome of *C. gloeosporioides* to explore the pathogenic mechanism. Of them, a two-LysM-containing protein encoding gene was named *Cg2LysM* (XM_045401316.1) and was amplified by RT-PCR. The open reading frame (ORF) of *Cg2LysM* was 486 bp which encoded a 162 amino acid polypeptide with a signal peptide (1–27aa) at its N-terminal and two conserved LysM domains (45–87aa and 115–158aa; Supplementary Figure S1). Fungal LysM proteins were classified into type A, B, C, D and E based on their overall domain architecture. Of them, type-A proteins were referred to as putative “LysM effectors” which might have a role in the infection process, type B proteins were homologous to chitinases, type C proteins were homologous to the carbohydrate-binding antiviral protein cyanovirin-N, type D proteins were predicted to be involved in chitin binding but their function is unknown, and type E proteins were putative N-acetylmuramoyl-L-alanine amidases (de Jonge and Thomma, 2009). In order to identify the type of Cg2LysM belonged to, a Neighbor joining tree was generated based on the amino acid sequences of Cg2LysM and different types of LysM proteins from fungal species (Figure 1). Phylogeny analysis revealed that Cg2LysM was clustered with type A of LysM proteins and was closest to the LysM proteins ChELP1 and ChELP2 of *Colletotrichum higginsianum*, indicating that Cg2LysM might play roles in the infection process of *C. gloeosporiordes* as an effector.

### *Cg2LysM* contributed to the virulence toward rubber tree leaves

**Figure 2:**
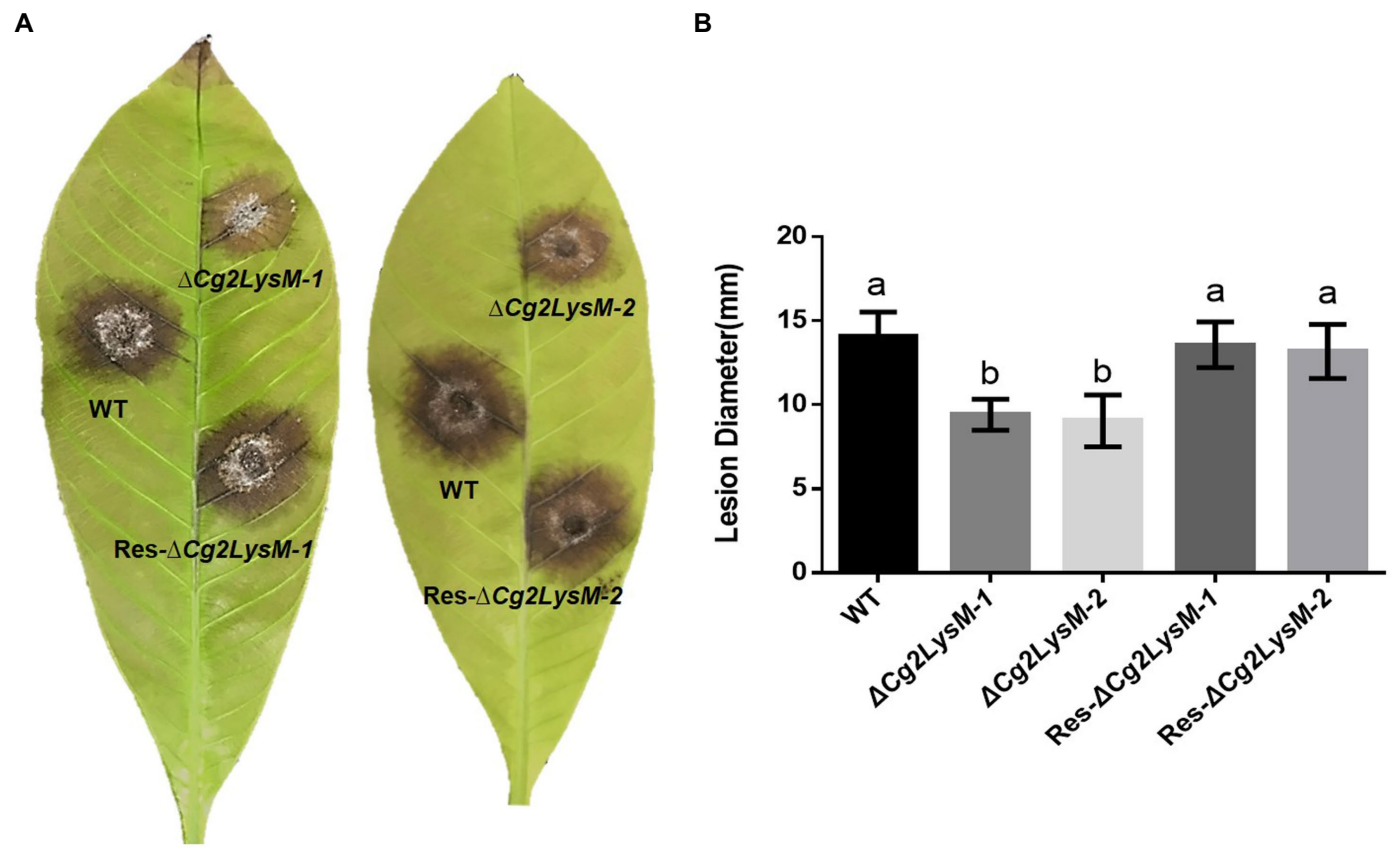
Pathogenicity test of WT, Δ*Cg2LysM* and Res-Δ*Cg2LysM* on rubber tree leaves. **(A)** Disease symptoms on rubber tree (*Hevea brasiliensis*) leaves at 4 days post inoculation with WT, Δ*Cg2LysM* and Res-Δ*Cg2LysM*. **(B)** Statistic analysis of lesion diameter at 4 days post inoculation with WT, Δ*Cg2LysM* and Res-Δ*Cg2LysM*. Data are shown as the means ± SD from three independent experiments and at least 20 infections per experiment. Different letters above columns indicate a significant difference (*p* < 0.05).

To investigate the biological roles of *Cg2LysM* in *C. gloeosporioides*, two *Cg2LysM* knockout mutants (∆*Cg2LysM-1 and* ∆*Cg2LysM-2*) were generated by gene replacement and PEG-mediated protoplast transformation. In the △*Cg2LysM-1 and* ∆*Cg2LysM-2*, the original *Cg2LysM* sequence was replaced by chlorimuron resistance cassettes (Supplementary Figure S2A). The complementary mutants (Res-∆*Cg2LysM-1 and* Res-∆*Cg2LysM-2*) were generated by introduced *Cg2LysM* into ∆*Cg2LysM-1 and* ∆*Cg2LysM-2* by hygromycine resistance cassette. ∆*Cg2LysM* mutants and Res-∆*Cg2LysM* mutants were examined by PCR (Supplementary Figures S2B–D). The virulence of wild type strain (WT), ∆*Cg2LysM* strains, and Res-∆*Cg2LysM* strains were examined with isolated rubber tree leaves. As showed in Figure 2A, typical necrotic lesions were observed on the rubber tree leaves at 4 days post inoculation with WT, ∆*Cg2LysM-1,* ∆*Cg2LysM-2*, Res-∆*Cg2LysM-1*, and Res-∆*Cg2LysM-2*. Statistical analysis showed that the size of necrotic lesions induced by two ∆*Cg2LysM* mutants were significantly larger than that induced by WT, and two Res-∆*Cg2LysM* mutants could restore the virulence of two ∆*Cg2LysM* strains, respectively (Figure 2B). These results suggested that *Cg2LysM* contributed to the virulence of *C. gloeosporioides* to rubber tree.

### Cg2LysM contributed to conidiation, appressorium formation, and invasion

**Figure 3:**
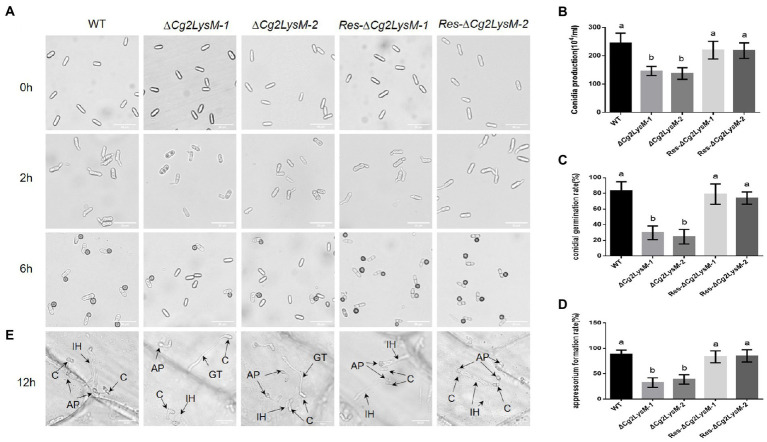
Conidial germination and appressorium formation assay of WT, Δ*Cg2LysM* and Res-Δ*Cg2LysM*. **(A)** Appressorium development progress of WT, ∆*Cg2LysM*, Res-∆*Cg2LysM*. **(B)** Conidia production of WT, ∆*Cg2LysM*, Res-∆*Cg2LysM* in CM media. Ten microscope fields were surveyed for every sample. **(C)** Conidia germination rates of WT, ∆*Cg2LysM*, and Res-∆*Cg2LysM* at 2 h time intervals. **(D)** Appressorium formation rates of WT, ∆*Cg2LysM*, Res-∆*Cg2LysM* at 6 h time intervals. **(E)** Invasion observation in onion epidermal cells. Equal volumes (5 μl) of conidial suspensions (2.5 × 10^5^ conidia/ml) from WT, ∆*Cg2LysM*, Res-∆*Cg2LysM* were inoculated on the onion epidermal cells at 12 h time intervals. C, AP, IH, GT indicates the conidia, appressorium, infection hyphae and germ tube, separately. Data are shown as the means ± SD from three independent experiments. Different letters above columns indicate a significant difference (*p* < 0.05). Bars = 20 μm.

To explore the possible roles of Cg2LysM in fungal growth and development, the fungal growth, conidiation ability, conidia germination and appressorium formation were analyzed. The aerial hyphae growth speed of WT, ∆*Cg2LysM* and Res-∆*Cg2LysM* strains showed no significant differences (Supplementary Figures S3A,B). Although the conidia morphology of ∆*Cg2LysM*-*1* and ∆*Cg2LysM-2* was unchanged compared with the WT and Res-∆*Cg2LysM*-*1* and Res-∆*Cg2LysM*-*2*, the conidia production was significantly reduced (Figures 3A,B). The conidia germination rate and appressorium formation rate of ∆*Cg2LysM-1* and ∆*Cg2LysM*-*2* were sharply reduced to approximately one-third that of the WT, and Res-*Cg2LysM-1* and Res-∆*Cg2LysM-2* could restore the conidiations of ∆*Cg2LysM-1* and ∆*Cg2LysM-2*, respectively (Figures 3C,D). In addition, the invasion processes of WT, ∆*Cg2LysM* and Res-∆*Cg2LysM* were observed on onions epidermis under optical microscope. Compared with WT and Res-∆*Cg2LysM* stains which could successfully complete infection and form normal primary hyphae in onions epidermis cells, two ∆*Cg2LysM* strains formed abnormal primary hyphae that failed to develop further (Figure 3E). These data indicated that *Cg2LysM* was involved in the regulation of conidiation, conidia germination, appressorium formation and invasion process.

### Cg2LysM was involved in melanin synthesis

**Figure 4:**
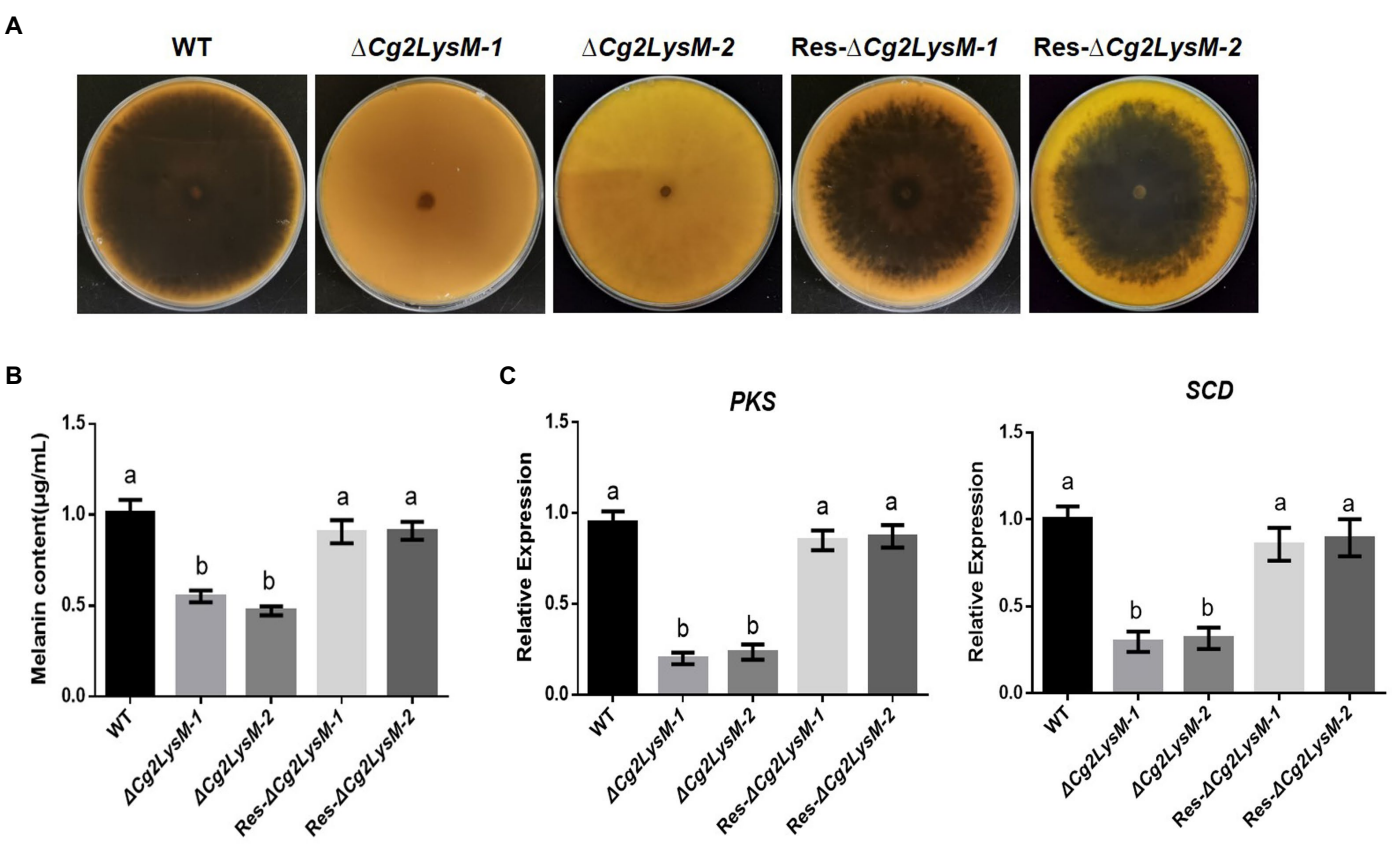
Melanin content assay of WT, Δ*Cg2LysM* and Res-Δ*Cg2LysM* strains. Colony morphology of WT, **(A)** Δ*Cg2LysM*, and Res-Δ*Cg2LysM* on PDA at 7 days. **(B)** Statistic analysis of melanin content of WT, Δ*Cg2LysM*, and Res-Δ*Cg2LysM*. **(C)** Relative expression assay of melanin synthesis gene *CgPKS1* and *CgSCD1*. Data are shown as the means ± SD from three independent experiments. Different letters above columns indicate a significant difference (*p* < 0.05).

When cultured on PDA medium plates, it was observed that ∆*Cg2LysM* colonies were significantly lighter in color than WT and Res-∆*Cg2LysM* colonies (Figure 4A). Quantitative analysis of melanin showed that the melanin contents of ∆*Cg2LysM*-*1* and ∆*Cg2LysM-2* were significantly lower than that of WT, and Res-∆*Cg2LysM-1* and Res-∆*Cg2LysM-2* restored melanin contents of ∆*Cg2LysM1* and ∆*Cg2LysM-2*, respectively (Figure 4B). A polyketide synthase gene *CgPks1* and a scytalone dehydratase gene *CgSCD1* were considered to be required for melanin synthesis in *C. gloeosporioide*s (Wang et al., 2020, 2021). Quantitative analysis results showed that the expression level of *CgPks1* and *CgSCD1* in ∆*Cg2LysM* were reduced to approximately one-second compared with that in WT and Res-∆*Cg2LysM* (Figure 4C), indicating the involvement of Cg2LysM in melanin synthesis of *C. gloeosporioides* be related to the modulation of *CgPks1* and *CgSCD1*.

### Cg2LysM had chitin-binding activity

**Figure 5:**
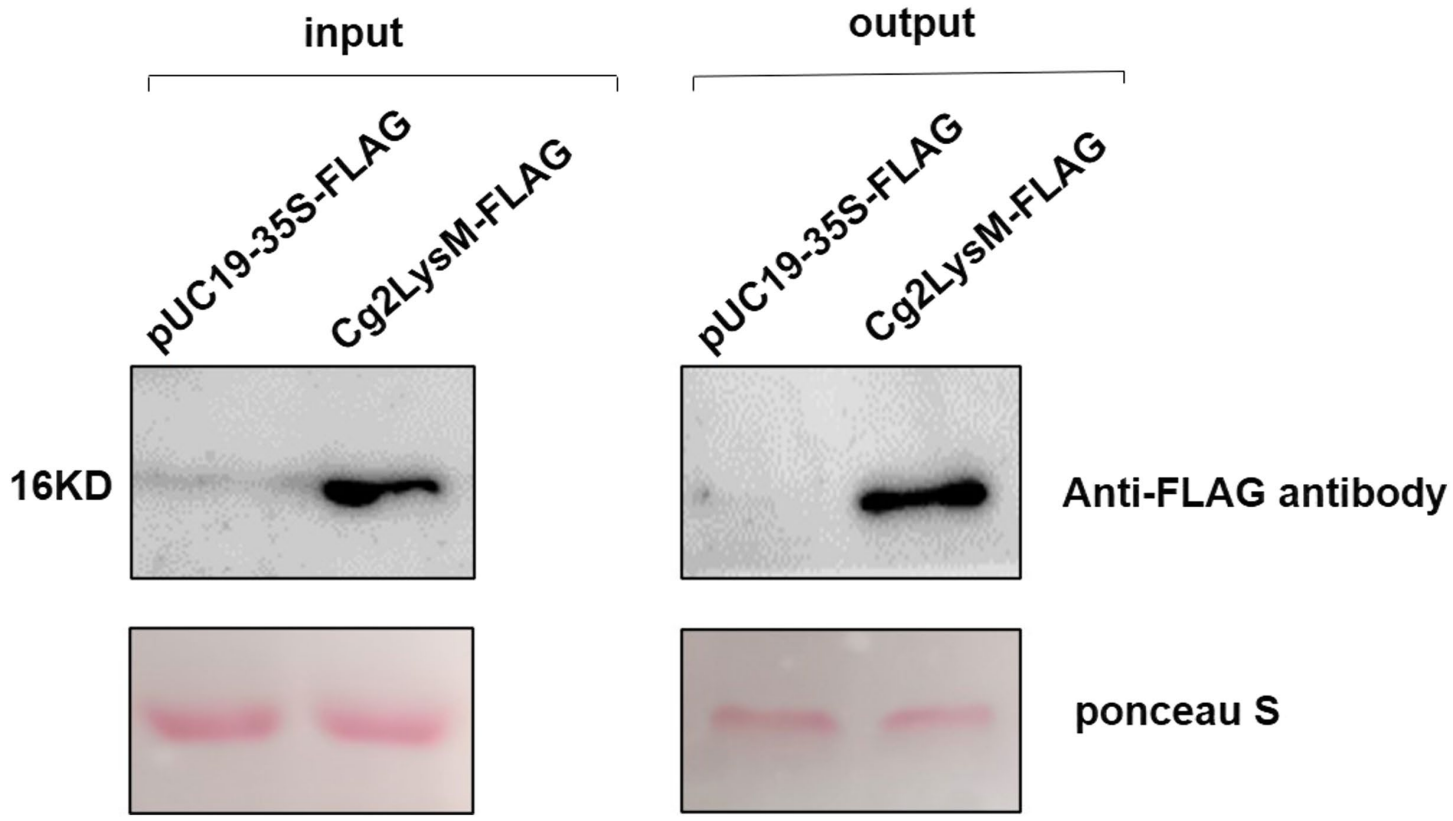
Analysis of chitin binding activity of Cg2LysM. Total protein extraction solutions from rubber tree mesophyll protoplasts expressing empty vector (control) and Cg2LysM were incubated with chitin beads, respectively. Chitin beads were boiled in phosphate buffer and analyzed by Western blot with primary antibody anti-flag antibody (1:10,000) and secondary FITC-conjugated goat antimouse antibody (1:5,000).

Several researches had demonstrated that LysM type effectors from some phytopathogenic fungi had chitin-binding activity which could block the chitin triggered innate immunity in plants (Jonge et al., 2010; Marshall et al., 2011; Kombrink et al., 2017; Dolfors et al., 2019). In order to determine the chitin-binding activity of Cg2LysM, Cg2LysM-FLAG fusion protein was transiently expressed in mesophyll protoplasts of rubber tree and was precipitated with chitin magnetic beads. A 16 kDa blot was detected with Anti-FLAG antibody in the eluent of chitin magnetic beads inoculated with total protein containing Cg2LysM-FLAG, but not in the control eluent (Figure 5), indicating that Cg2LysM protein was able to bind with chitin.

### Cg2LysM suppressed chitin-triggered ROS production

**Figure 6:**
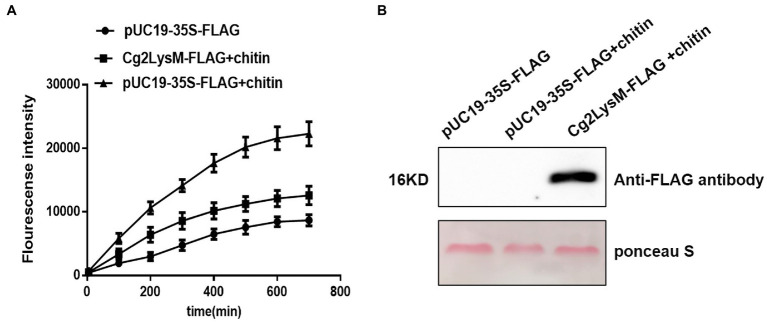
Effector of Cg2LysM on the ROS production induced by chitin. **(A)** ROS assay in rubber tree mesophyll protoplasts expressing empty vector without/with chitin treatment and expressing Cg2LysM with chitin treatment. ROS contents was measured by DCFH-DA. **(B)** Expression assay of Cg2LysM in different samples. Expression level of Cg2LysM was detected by western blotting.

Rapid and transient accumulation of ROS is one of the best characterized events in early PAMP-signaling of plant innate immune (Smith and Heese, 2014; Stael et al., 2015). Chitin could induce ROS accumulation in rubber tree mesophyll protoplasts (Zhang et al., 2016). Considering the chitin-binding activity of Cg2LysM, the effect of Cg2LysM on chitin-triggered ROS production in rubber tree mesophyll protoplasts were measured using H2DCFDA (dichlorofluorescin diacetate) staining. As showed in Figure 6, in the rubber tree mesophyll protoplasts harboring pUC19-35S-Flag vector, ROS production increased slowly within 700 min of transient expression. When the rubber tree mesophyll protoplasts transiently expressing pUC19-35S-Flag vector were treated with chitin, the ROS production increased sharply within 700 min. Further, when the rubber tree mesophyll protoplasts expressing Cg2LysM-Flag fusion protein were treated with chitin, the ROS level of which was significantly lower than that of protoplasts harboring pUC19-35S-Flag vector with chitin treatment, suggesting that Cg2LysM could suppress chitin-triggered ROS production.

### Cg2LysM changed the transcripts of defense-related genes

**Figure 7:**
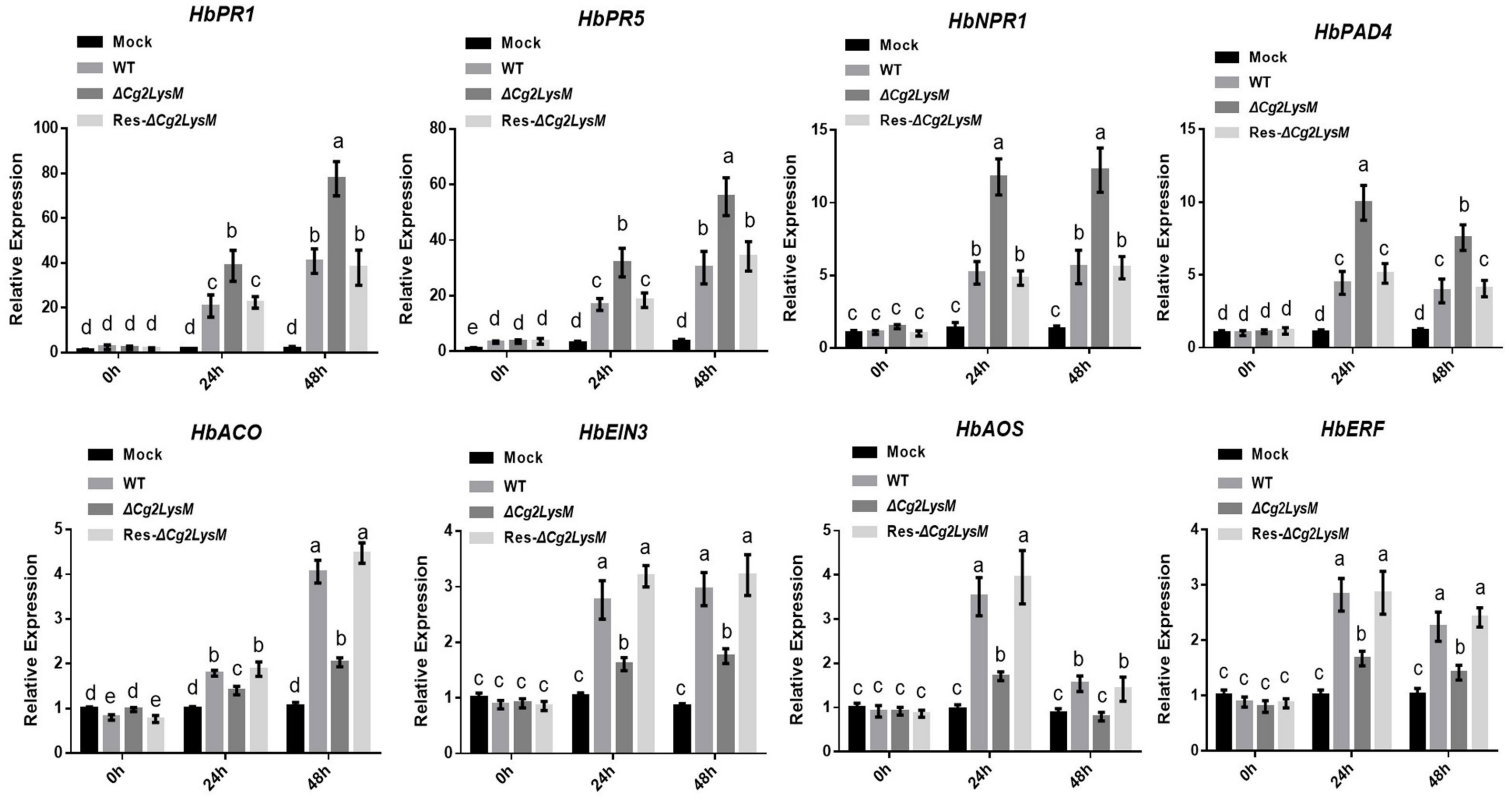
Relative expression assay of defense related genes in rubber tree leaves inoculated with WT, Δ*Cg2LysM*, and Res-Δ*Cg2LysM* strains. Data are shown as the means ± SD from three independent experiments. Different letters above columns indicate a significant difference (*p* < 0.05).

The expression profiles of defense-related genes *HbPR1, HbPR5, HbNPR1, HbPAD4, HbACO, HbEIN3, HbAOS,* and *HbERF* were analyzed by qRT-PCR in rubber tree leaves inoculated with WT, ∆*Cg2LysM* and Res-∆*Cg2LysM,* respectively. As showed in Figure 7, the different expressions of these defense related genes induced by inoculation with WT and Res-∆Cg2LysM were not obvious. The expression levels of *HbPR1*, *HbPR5, HbNPR1,* and *HbPAD4* were increased significantly at 24 h post inoculated with ∆*Cg2LysM* compared to that inoculated with WT. However, the expressions of *HbACO, HbEIN3, HbAOS,* and *HbERF* were inhibited significantly at 24 h post inoculated with ∆*Cg2LysM* compared to that inoculated with WT. *HbPR1, HbPR5, HbNPR1,* and *HbPAD4* were considered to be involved in salicylic acid (SA) biosynthesis and defense response (Gruner et al., 2013; Cui et al., 2017), *HbAOS* and *HbERF* in ethylene (JA) synthesis and signaling regulation (Laudert and Weiler, 1998; Ruan et al., 2019), *HbACO* and *HbEIN3* in jasmonic acid (ET) biosynthesis and signaling regulation (Wang et al., 2002; Dolgikh et al., 2019). Therefore, these results showed that the absent of Cg2LysM enhanced SA-mediated defense response but inhibited JA-/ET-mediated defense response in rubber tree.

## Discussion

Multiple LysM-containing proteins were found in both prokaryotes and eukaryotes. Many fungal pathogens encoded effectors containing LysM domain (de Jonge and Thomma, 2009). Based on evolutionary analyses, LysM domains were classified into two groups: fungal-specific group and fungal/bacterial group (Akcapinar et al., 2015). The LysM motif of fungal/bacterial group consisted of about 40 amino acids with none or only one cysteine, e.g., those of *Cladosporium fulvum* Ecp6, *Mycosphaerella graminicola* Mg3LysM and *Magnaporthe oryzae* Slp1(Bolton et al., 2008; Marshall et al., 2011; Mentlak et al., 2012); the LysM domain of fungal specific group consisted of 53 amino acids with four conserved cysteines (positions 5, 17, 42 and 52) and an N in a WNP (positions 35–37), e.g., those of *Tricho derma atroviride* Tal6 (Seidl-Seiboth et al., 2013). Based on their overall domain architecture, fungal LysM proteins were classified into five types. Of them, the members of Type A had a signal peptide and did not contain any recognizable motif other than a varying number of LysMs, which were referred to as “LysM effectors” and might play roles in the infection process; However, Type B-E contained not only one or more LysM but also other recognizable conserved domains such as Cys-rich chitin-binding domain, enzymatic domain, CyanoVirin-N homology domain, chitin recognition domain and so on (de Jonge and Thomma, 2009). In this study, Cg2LysM had two LysM motifs of about 40-aa (46–87aa and 115–158aa), the former LysM motif containing a cysteine (position at 65aa) and the latter without cysteine (Supplementary Figure S1A), which was consist with the features of fungal/bacterial group of LysM-containing proteins. Cg2LysM had only a recognizable N terminal signal peptide (1–27aa) in addition to two LysM motifs (Supplementary Figure S1A), and Cg2LysM was clustered with type I of known LysM proteins in phylogenetic tree, indicating that Cg2LysM was a A-type LysM effector protein.

As pathogenic factor, some LysM effector proteins contributed to fungal virulence and regulation of fungal growth and development. In *Cladosporium fulvum*, RNAi-mediated silencing of Ecp6 resulted in a clearly delayed progression of disease on tomato plants, and heterologous expression of Ecp6 in *Fusarium oxysporum* f. sp. *lycopersici* enhanced virulence on tomato (Bolton et al., 2008). In *Mycosphaerella graminicola*, Mg3LysM deletion mutant showed only weak virulence toward wheat leaves companying with a complete lack of necrotic lesions bearing pycnidia and a dramatically reduced fungal biomass (Marshall et al., 2011). In *Magnaporthe oryzae*, deletion of a LysM effector protein SLP1 significantly reduced the ability to cause disease, which was associated with a reduced proliferate ability of *slp1* deletion mutant in host tissues (Mentlak et al., 2012). In *C. higginsianum*, LysM effector protein ChELP1 and ChELP2 were required for virulence and ChELP1 RNAi mutants displayed abnormal appressorium in morphology with impaired penetration ability (Takahara et al., 2016). In *Verticillium dahliae*, LysM effector protein Vd2LysM, but not Vd4LysM, Vd5LysM and Vd6LysM, contributed to virulence on tomato (de Jonge et al., 2013; Kombrink et al., 2017). A LysM protein RsLysM played an important role in *Rhizoctonia solani* virulence, and heterologous expression of RsLysM in *Cercospora beticola* increased fungal colonization ability and necrotic lesion size on host (Dolfors et al., 2019). In plant-beneficial fungus *Trichoderma atroviride*, a seven LysMs effector TAL6 specifically inhibited the germination of *Trichoderma* spp. and increased the fungus mycoparasitic capacity (Seidl-Seiboth et al., 2013; Romero-Contreras et al., 2019). In *Penicillium expansum*, PeLysM3 null mutant exhibited slightly lower rate of radial growth, significantly lower percent of germinating spores and shorter germ tubes (Levin et al., 2017). In *M. graminicola*, LysM effectors regulated pycnidia formation on wheat (Tian et al., 2021). Based on our data, Cg2LysM deletion mutant showed impaired virulence toward rubber tree (Figure 2). Moreover, Cg2LysM deletion resulted in significant reduced conidia production, conidia germination, appressorium formation of *C. gloeosporioides*, and abnormal primary hyphae which failing to finish the invasion process (Figure 3). These findings demonstrated that LysM effectors could contribute to fungal virulence through regulation of fungal growth and development, including the development of invasion structure.

Chitin is the major component of fungal cell walls and acts as PAMPs which trigger PTI in plant cells (Jones and Dangl, 2006). Most fungal pathogens evaded host immunity through inhibiting chitin-triggered immunity by blocking chitin sensing or signaling (Hu et al., 2021). Chitin-triggered immunity is known to result in induction of ROS burst and pathogenesis-related genes (Mentlak et al., 2012). LysM domain had been demonstrated to have chitin binding activity (Bolton et al., 2008; Jonge et al., 2010). The function of LysM effectors relied on the binding properties of the LysM domain to the fungal chitin to evade host immunity (Kombrink et al., 2011; Marshall et al., 2011). Ecp6 of *C. fulvum* suppressed chitin-triggered ROS burst in host cells (Jonge et al., 2010). Mg3LysM of *M. graminicola* inhibited the expression of Pathogenesis-Related (PR) gene and chitinase gene in wheat at the later stage of infection (Marshall et al., 2011). LysM effectors MbLysM5 and MbLysM19 of *Marssonina brunnea* weakened the expression of PDF1.2 which was chitin-induced in *Arabidopsis thaliana* (Jiang et al., 2014). SLP1 of *M. oryzae* prevented the expression of rice Phe ammonia lyase gene, *PAL1*, and the β-glucanase-encoding gene, *rBG* (Mentlak et al., 2012). Our group had reported that chitin-triggered immune responses included transcriptional reprogramming of defense-related genes *HbPR1* and *HbPR5* and ROS accumulation in mesophyll protoplasts of rubber tree (Zhang et al., 2016). In this study, Cg2LysM also showed chitin-binding activity (Figure 5), and the presence of Cg2LysM suppressed chitin-triggered ROS production and the expression of defense-related genes *HbPR1* and *HbPR5* in rubber tree mesophyll protoplasts (Figures 6, 7). These data confirmed that Cg2LysM, like other LysM effectors, could contribute to invasion through manipulating chitin-triggered plant immunity.

Phytohormones salicylic acid (SA), jasmonic acid (JA), and ethylene (ET) were known as classical primary defense hormones which played roles in the regulation of basal resistance against multiple pathogens (Bari and Jones, 2009; Robert-Seilaniantz et al., 2011; Pieterse et al., 2012). SA contributed to PTI and ETI, and exogenous SA application induced pathogenesis related (PR) genes expression, endogenous SA levels as well as increased disease resistance (Yang et al., 2015). Phytoalexin Deficient4 (PAD4) was involved in SA accumulation by promoting principal SA biosynthetic enzyme Isochorismate synthase1 (ICS1) gene expression (Cui et al., 2017). 1-aminocyclopropane-1-carboxylic acid (ACC) synthase (ACS) and 2-oxoglutarate ACC oxidase (ACO) were two key enzymes in ET biosynthesis (Wang et al., 2002). EIN3/EIL1 transcription factors were known as the key regulators of ethylene signaling that sustained a variety of plant responses to ethylene (Dolgikh et al., 2019). Allene oxide synthase (AOS) was a major control point for JA biosynthesis in *Arabidopsis thaliana* and transcriptional factor Ethylene-Responsive Factor (ERF) was an important component of ET signaling pathway (Laudert and Weiler, 1998; Wasternack and Song, 2017). There are complex interactions among various plant hormone signaling pathways. Despite the complex interaction among SA, JA, and ET defense pathways, the antagonistic interaction between SA and JA, and the synergistic interaction between JA and ET seemed to be dominant (Yang et al., 2015). In this study, the expression profiles of some genes involved in the SA, JA, ET defense response signaling pathway were analyzed in rubber tree leaves inoculated with WT, ∆*Cg2LysM* and Res-∆*Cg2LysM,* respectively. The results showed that the genes involved in SA defense signaling (*HbPR1, HbPR5, HbNPR1,* and *HbPAD4*) were induced significantly at 24 h in rubber tree leaves post inoculated with ∆*Cg2LysM* compared to that inoculated with WT and Res-∆Cg2LysM, but the genes involved in both JA and ET defense signaling (*HbAOS, HbERF*, *HbACO,* and *HbEIN3*) were repressed in rubber tree leaves post inoculated with ∆*Cg2LysM* compared to that inoculated with WT and Res-∆Cg2LysM (Figure 7). These data indicated that Cg2LysM negatively regulated SA mediated defense pathway and positively regulates JA and ET mediated signaling pathway. However, the regulatory mechanism remains unclear.

In fungi, melanin was one of the well-known secondary metabolites which frequently was found in appressorium, a specialized invasion structurer (Bechinger et al., 1999). Melanin played a critical role in pathogenicity through mediating the buildup of high pressure in the appressorium providing the essential driving force for mechanical penetration (Kaverinathan et al., 2017). In our case, we found that the melanin content of ∆*Cg2LysM* was significantly lower than that of WT (Figures 4A,B). This is the first report on the function of LysM effector in fungal melanin. In *Colletotrichum gloeosporioides*, a polyketide synthase CgPks1 and a scytalone dehydratase CgSCD1 were involved in melanin synthesis and pathogenicity, and CgPks1 contributed to turgor pressure of the appressorium, but not CgSCD1 (Wang et al., 2020, 2021). Here we detected the transcriptional expression of CgPks1 and CgSCD1 in ∆*Cg2LysM*, the result showed that level of CgPks1 and CgSCD1 in ∆*Cg2LysM* was significantly lower than that in WT (Figure 4C), indicating that Cg2LysM modulated melanin synthesis through CgPks1 and CgSCD1. Considering that Cg2LysM is required for successful infection of *C. gloeosporioides* on onion epidermal cells (Figure 3E), we speculate that the regulation of Cg2LysM on virulence may be related to the development of infection structure and infection process involved in melanin.

In summary, our results demonstrated the function of a LysM effector Cg2LysM on pathogenicity of *C. gloeosporioides* toward rubber tree through not only self-regulation of fungal conidiation, appressorium formation and melanin mediated infection process, but also manipulation of rubber tree immune responses such as ROS production and transcriptional programs of defense-related genes *HbPR1* and *HbPR5*.

## Data availability statement

The original contributions presented in the study are included in the article/Supplementary material, further inquiries can be directed to the corresponding authors.

## Author contributions

LZ carried out most of the experiments and analyzed the data. LF cloned the *Cg2LysM* gene. ZL did the vector construction. QW helped to finish the microscope observation. LZ, BA, and HL wrote the manuscript. CH revised the manuscript. All authors contributed to the article and approved the submitted version.

## Funding

This study was supported by the National Natural Science Foundation of China (Grant Nos. 32060591 and 32260710).

## Conflict of interest

The authors declare that the research was conducted in the absence of any commercial or financial relationships that could be construed as a potential conflict of interest.

## Publisher’s note

All claims expressed in this article are solely those of the authors and do not necessarily represent those of their affiliated organizations, or those of the publisher, the editors and the reviewers. Any product that may be evaluated in this article, or claim that may be made by its manufacturer, is not guaranteed or endorsed by the publisher.

## Footnotes

^1^ http://www.cbs.dtu.dk/services/SignalP/

^2^ http://smart.embl-heidelberg.de/

## Supplementary material

SUPPLEMENTARY FIGURE S1 Nucleotide sequence and amino acid protein sequence of Cg2LysM. The underlined part is the amino acid sequence of the signal peptide region. The black and gray shaded part indicate two Lysin motif position of Cg2LysM. *indicates a stop codon.

SUPPLEMENTARY FIGURE S2 Generation and molecular confirmation of *Cg2LysM* knockout mutant (∆*Cg2LysM*) and complementary mutants (Res-∆*Cg2LysM*). **(A)** Schematic diagram of ∆*Cg2LysM* and Res-∆*Cg2LysM*. **(B, C)** Diagnostic PCR analysis for correct integration of 5’ and 3’ recombinant fragment into *Cg2LysM* locus. **(D)** Determination of *Cg2LysM* insertion into the genome of ∆*Cg2LysM* and Res-∆*Cg2LysM* by PCR.

SUPPLEMENTARY FIGURE S3 Growth rate assays of ∆*Cg2LysM* and Res-∆*Cg2LysM*. **(A)** Colonial morphology of WT, ∆*Cg2LysM* and Res-∆*Cg2LysM* on PDA medium at 1, 3, 5, 7 days. **(B)** Statistic analysis of colony diameters of WT, ∆*Cg2LysM* and Res-∆*Cg2LysM* on PDA at 1, 3, 5, 7 days. Error bars indicated standard deviation, and columns with different letters indicate significant difference (*p*<0.05).

SUPPLEMENTARY TABLE S1 Primers used in the present study.
