# Supplementary figures and images for: Colletotrichum gloeosporioides Cg2LysM contributed to virulence toward rubber tree through affecting invasive structure and inhibiting chitin-triggered plant immunity
Source: Front Microbiol. 2023 Feb 17;14:1129101. doi: 10.3389/fmicb.2023.1129101 (PMC9982014; doi:10.3389/fmicb.2023.1129101)

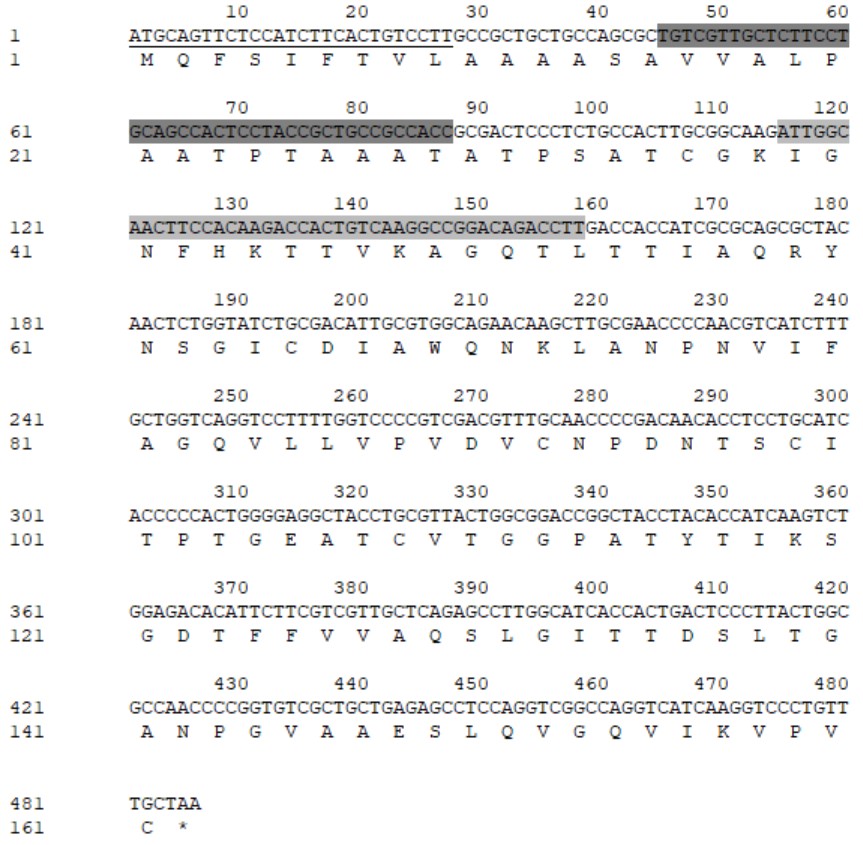

Supplement: SUPPLEMENTARY FIGURE S1 — Nucleotide sequence and amino acid protein sequence of Cg2LysM. The underlined part is the amino acid sequence of the signal peptide region. The black and gray shaded part indicate two Lysin motif position of Cg2LysM. *indicates a stop codon. [file Image_1.JPEG]

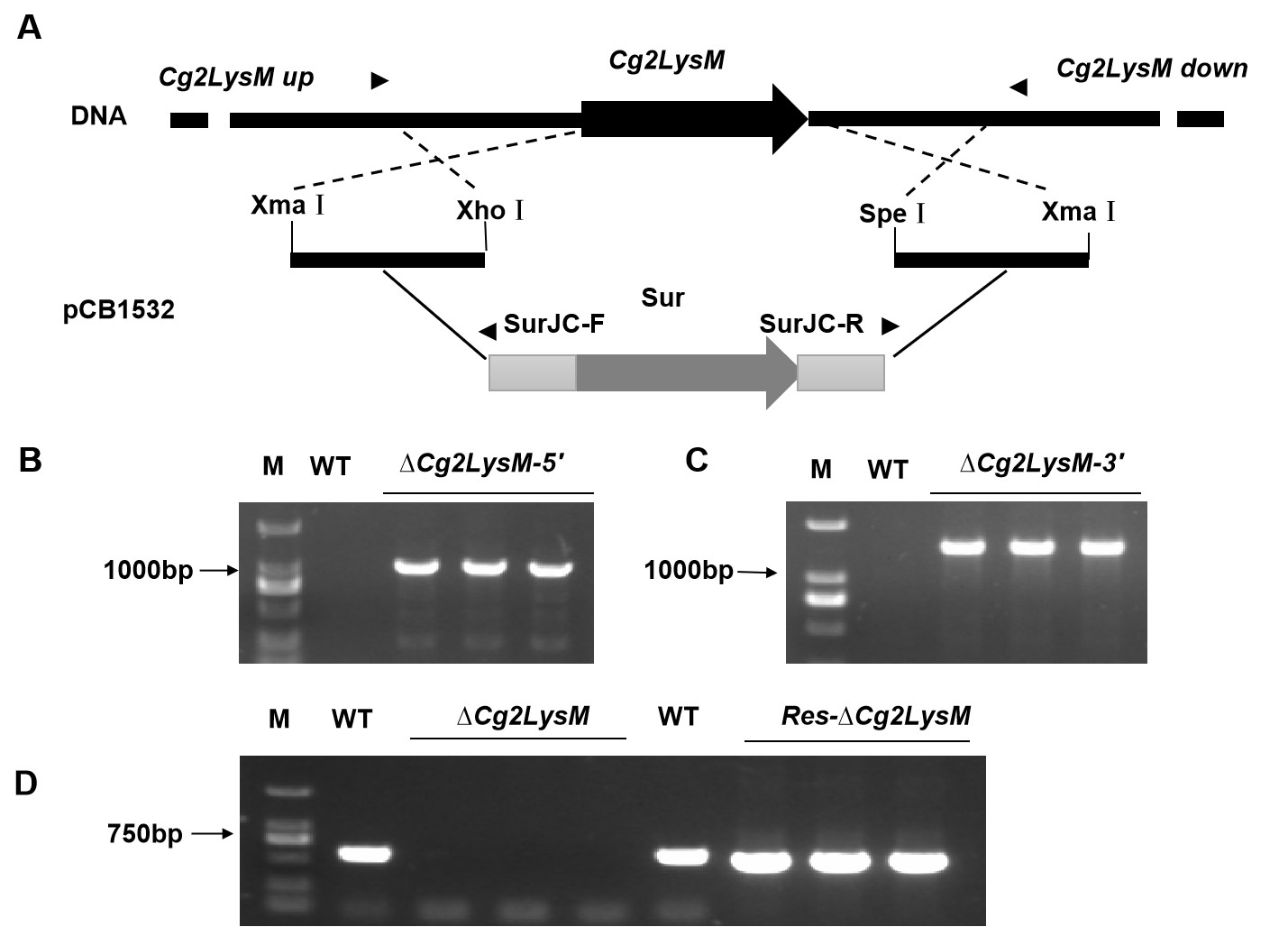

Supplement: SUPPLEMENTARY FIGURE S2 — Generation and molecular confirmation of Cg2LysM knockout mutant (∆Cg2LysM) and complementary mutants (Res-∆Cg2LysM). (A) Schematic diagram of ∆Cg2LysM and Res-∆Cg2LysM. (B, C) Diagnostic PCR analysis for correct integration of 5’ and 3’ recombinant fragment into Cg2LysM locus. (D) Determination of Cg2LysM insertion into the genome of ∆Cg2LysM and Res-∆Cg2LysM by PCR. [file Image_2.JPEG]

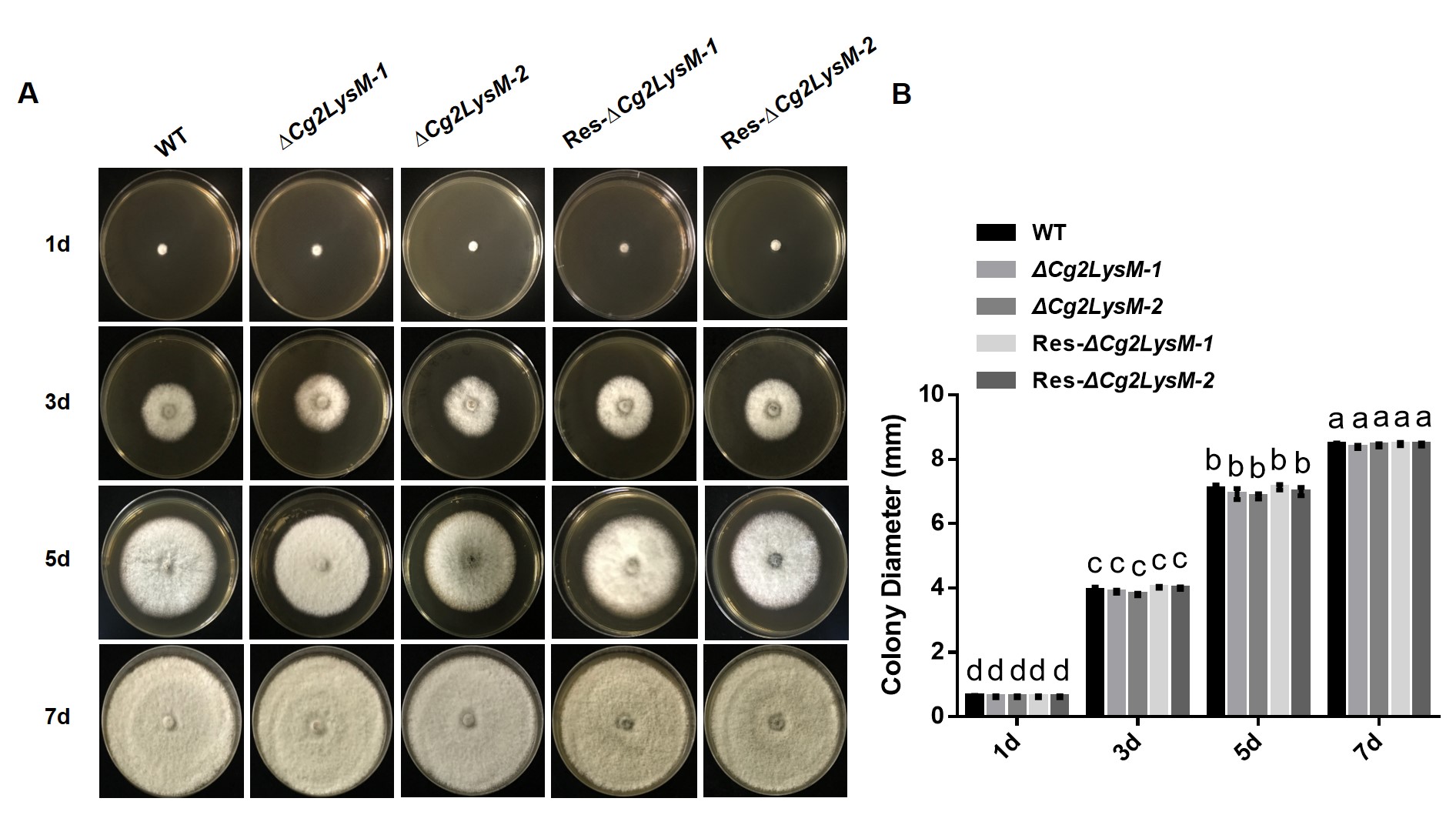

Supplement: SUPPLEMENTARY FIGURE S3 — Growth rate assays of ∆Cg2LysM and Res-∆Cg2LysM. (A) Colonial morphology of WT, ∆Cg2LysM and Res-∆Cg2LysM on PDA medium at 1, 3, 5, 7 days. (B) Statistic analysis of colony diameters of WT, ∆Cg2LysM and Res-∆Cg2LysM on PDA at 1, 3, 5, 7 days. Error bars indicated standard deviation, and columns with different letters indicate significant difference (p<0.05). [file Image_3.JPEG]
